# *Salmonella* infection induces the reorganization of follicular dendritic cell networks concomitant with the failure to generate germinal centers

**DOI:** 10.1016/j.isci.2023.106310

**Published:** 2023-03-02

**Authors:** Edith Marcial-Juárez, Marisol Pérez-Toledo, Saba Nayar, Elena Pipi, Areej Alshayea, Ruby Persaud, Sian E. Jossi, Rachel Lamerton, Francesca Barone, Ian R. Henderson, Adam F. Cunningham

**Affiliations:** 1 Institute of Immunology and Immunotherapy, University of Birmingham, Birmingham, West Midlands, B15 2TT, United Kingdom; 2 Rheumatology Research Group, Institute of Inflammation and Ageing, University of Birmingham, Birmingham, West Midlands, B15 2TT, United Kingdom; 3 National Institute for Health Research (NIHR) Birmingham Biomedical Research Centre, University Hospitals Birmingham NHS Foundation Trust, UK and Sandwell and West Birmingham Trust, Birmingham, West Midlands, B15 2TH, United Kingdom; 4 Institute for Molecular Bioscience, University of Queensland, Brisbane, QLD4072, Australia

**Keywords:** Immunology, Immune response, Components of the immune system, Microbiology, Cell biology

## Abstract

Germinal centers (GCs) are sites where plasma and memory B cells form to generate high-affinity, Ig class-switched antibodies. Specialized stromal cells called follicular dendritic cells (FDCs) are essential for GC formation. During systemic *Salmonella* Typhimurium (STm) infection GCs are absent, whereas extensive extrafollicular and switched antibody responses are maintained. The mechanisms that underpin the absence of GC formation are incompletely understood. Here, we demonstrate that STm induces a reversible disruption of niches within the splenic microenvironment, including the T and B cell compartments and the marginal zone. Alongside these effects after infection, mature FDC networks are strikingly absent, whereas immature FDC precursors, including marginal sinus pre-FDCs (MadCAM-1+) and perivascular pre-FDCs (PDGFRβ+) are enriched. As normal FDC networks re-establish, extensive GCs become detectable throughout the spleen. Therefore, the reorganization of FDC networks and the loss of GC responses are key, parallel features of systemic STm infections.

## Highlights

• Germinal centers (GC) do not form until *Salmonella* infection is largely resolved
• Infection perturbs splenic white pulp microarchitecture and cellular organization
• Follicular dendritic cell (FDC) networks are lost early during *Salmonella* infection
• Pre-FDC-like cells increase before the return of FDC networks and GC development

## Graphical abstract

**Figure:**
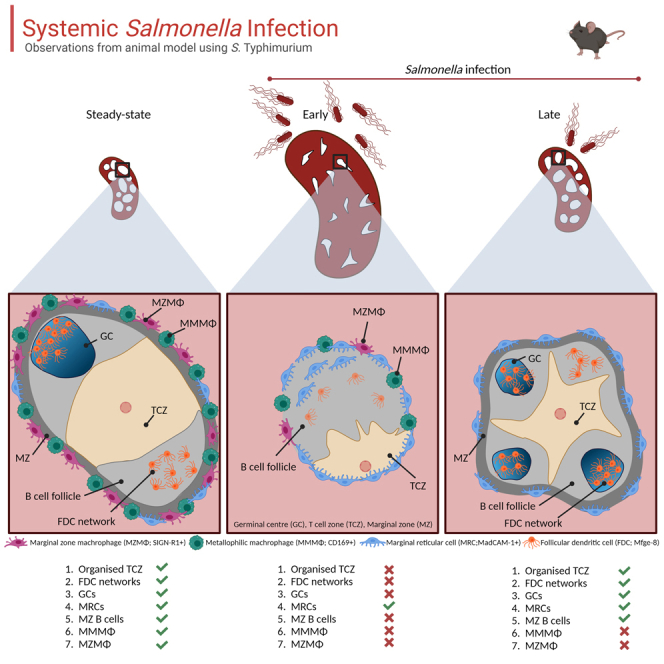

## Introduction

A hallmark of the mammalian immune response is the induction of adaptive immune responses to pathogens. An important contribution to the protection provided by the adaptive immune response is the generation of antibodies, which is a typical consequence of infection and a key aim of vaccination.^1^ Antibodies can be generated through two predominant, interconnected pathways. In primary responses, extrafollicular (EF) responses, which develop in the red pulp of the spleen or the medulla in lymph nodes, provide the first wave of IgM and IgG, and these antibodies are typically of modest affinity because there is limited affinity maturation of B cells that enter this pathway.^2^ Moreover, antibodies from plasma cells generated through the EF pathway is typically supplanted after a week^3^ or so by antibodies secreted by plasma cells derived from the germinal center (GC) response.^4^ GCs form in the B cell follicles of secondary lymphoid organs (SLOs) such as the spleen or lymph nodes.^5^^,^^6^ A key difference between the EF and GC responses is that antibodies generated from the GC tend to be of higher affinity and most memory B cells and the longest lived plasma cells derive from this response.^7^

The generation of these productive outputs from the GC requires the interplay of multiple cell types at different sites within SLO, with all processes dependent on the microarchitecture of SLO, including interactions between T and B cells in the T zones and the follicles.^8^^,^^9^ Although the organization of cells within T cell zones (TCZ) is dependent on CCL21/CCL19 secreted by fibroblastic reticular cells (FRC),^10^^,^^11^ the generation of B cell follicles relies on CXCL13 secreted by follicular dendritic cells (FDCs) and marginal reticular cells (MRCs).^12^^,^^13^^,^^14^ Moreover, FDCs play a critical function in the GC by holding antigens in their native conformation on their surface as immune complexes driving the selection of B cell clones with the highest affinity.^15^^,^^16^^,^^17^^,^^18^ Therefore, the organization of lymphoid tissues is essential for the efficient generation of productive immune responses and FDCs are crucial for normal follicle architecture and the GC reaction.^8^^,^^19^

Antibody responses induced during natural infection can help moderate the spread of the pathogen and secondary superinfections.^20^ Nevertheless, many pathogens can modulate the capacity to mount antibody responses during infection, potentially affecting the capacity of the host to deal with current or later infectious threats.^21^ Bacterial and parasitic infections such as those caused by *Ehrlichia muris*, *Salmonella* Typhimurium (STm) and *Plasmodium* spp. induce atypical GC responses^22^^,^^23^^,^^24^^,^^25^ and severe SARS-CoV-2 infections have also been shown to modulate the host’s capacity to induce GC.^26^ Indeed, in models of STm infection, GCs are not detectable until a month after infection, after which there is a significant increase of high-affinity antibodies in serum.^24^^,^^27^^,^^28^ The altered kinetics described for these pathogens are markedly different from the kinetics and processes described for GC responses that develop to non-viable, proteinaceous T-dependent antigens such as alum-precipitated ovalbumin or chicken gamma globulin.^29^^,^^30^ In these models, the GC response is established by a week after immunization, and the process is complete by around five weeks after immunization.^31^ Moreover, pathogens such as STm can impair the induction of GC responses to co-immunized antigens, demonstrating that this effect is not restricted to bacteria-associated antigens.^32^^,^^33^^,^^34^ The reasons for this delay in GC induction are incompletely understood but is not likely because of a failure to induce STm-specific T and B cell responses.^24^^,^^35^^,^^36^ For instance, within 24 h of STm infection, T cell priming and Th1 cell differentiation is already established, and extensive EF IgM and IgG antibody responses are detectable soon after infection.^24^^,^^33^ Notwithstanding these gaps in our understanding, some insights into why STm has these effects have been reported. Factors that influence the level of the GC response include the number of viable bacteria present,^24^^,^^28^ reduced T follicular helper (Tfh) cell differentiation by IL-12 mediated induction of T-bet,^37^ the recruitment of Sca-1+ monocytes to lymphoid organs and ineffective cellular respiration dependent on TNF and IFNγ.^34^ Despite these insights, it is unclear whether STm infection influences stromal cell populations and the compartments within SLO after infection. Here, we assessed the topological changes in the spleen and investigated the role of FDCs in the lack of GCs during acute STm infection and found that STm infection can modulate mature FDC networks for weeks and only when FDC networks reform do GC develop.

## Results

### *Salmonella* Typhimurium infection perturbs the organization of the white pulp microarchitecture

**Figure 1:**
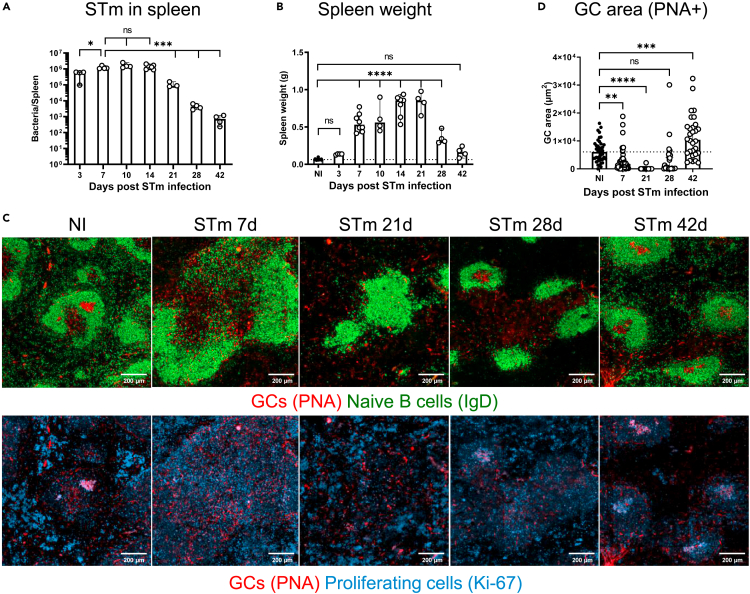
Kinetics of GC development during STm infection Mice were infected i.p. with 5 × 10^5^ STm SL3261 and the spleens recovered at the indicated times. (A) Bacteria loads (CFU) per spleen. Each symbol represents one mouse. (B) Spleen weights from individual infected and NI mice. (C) Top row: representative images of spleen sections from infected and NI mice stained to detect naive B cells (IgD+; green) and PNA-binding cells (red); bottom row: the same area as per top row showing PNA-binding cells (red) and proliferating cells (Ki-67+; blue). Scale bars 200 μm. (D) Quantification of GC area determined by measuring 45 follicles per section. Each point represents the area measured for each GC. Data is representative of 2–3 experiments with 4 mice each, the bar height represents the median, and the error bars display the 95% CI. One-way ANOVA and Tukey’s multiple comparison test for A or Dunnett’s multiple comparison test for B and D were performed. ∗p< 0.05, ∗∗p< 0.01, ∗∗∗p< 0.001, ∗∗∗∗p< 0.0001. ns, nonsignificant.

**Figure 2:**
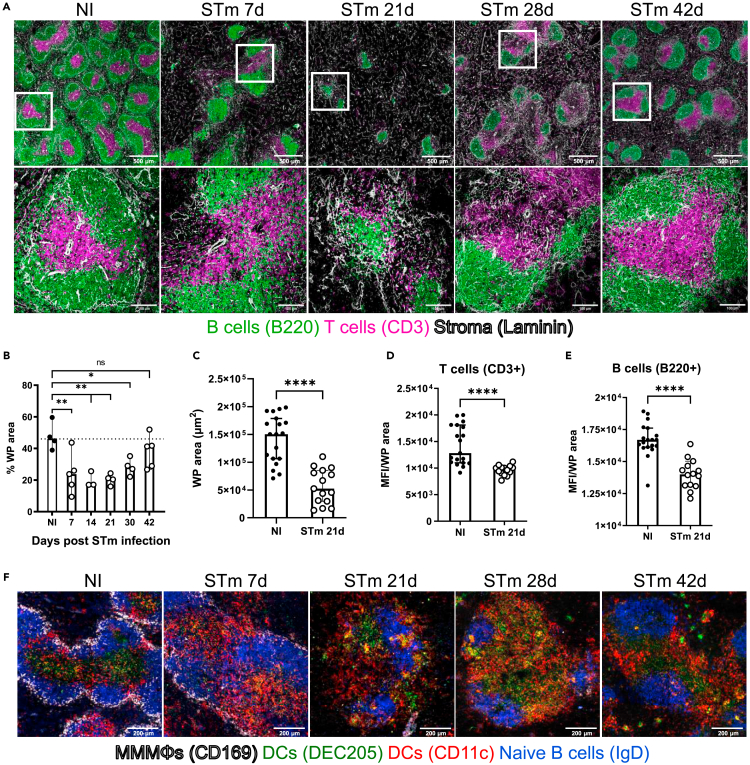
STm infection-induced alteration of the splenic microarchitecture Mice were infected as per Figure 1. (A) Cryosections from spleens were stained to detect T cells (CD3^+^; magenta), B cells (B220+; green), and the reticular fiber network (basement membrane, laminin+, white) to define the compartments in the WP. Representative low-magnification images are shown in the top row (scale bar 500 μm), and confocal images in the bottom row represent higher magnifications of the selected areas. Scale bar 100 μm. (B) Graph showing the proportion of WP per total area of spleen section. Each symbol represents a single mouse. (C) Graph showing the area (in μm^2^) of 15 randomly selected individual WP. (D and E) The density of B and T cell staining per WP (determined as the median fluorescence intensity (MFI) of the signal for B220 and CD3, respectively). (F) Cryosections from spleens were stained to detect MMMΦs (CD169+; white), DCs (DEC205+ in green and CD11c+ in red), and naive B cells (IgD+; blue). Scale bar 200 μm. Images and *in situ* quantification are representative of 2 experiments with at least 4 mice in each group; the bar height represents the median, and the error bars display the 95% CI. One-way ANOVA and Dunnett’s multiple comparison test for B, and two-tailed, unpaired, *t*-test for C-E was performed. ∗p< 0.05, ∗∗p< 0.01, ∗∗∗∗p< 0.0001. ns, nonsignificant.

Systemic infection of susceptible C57BL/6 mice with attenuated STm SL3261 results in a self-resolving infection characterized by rapid colonization of the spleen and bacterial numbers peaking from the first week before gradually decreasing after the third week (Figure 1A). In parallel, STm infection induces a marked splenomegaly which peaked at 21 days, when spleens were around 10 times the mass of non-infected control mice (Figure 1B). As reported previously,^24^ GCs are not a feature of early STm infections and are only consistently detected at day 42 after infection when the infection and associated splenomegaly has largely resolved (Figures 1C and 1D). We hypothesized that GC responses are delayed during STm infection because infection induces a perturbed white pulp (WP) topography thus inhibiting the generation of productive responses. Assessment of the WP containing the combined T and B cell compartments in the spleen showed that the proportion of the spleen that is WP decreased between 7 and 30 days after infection before recovering to similar proportions as non-infected mice by day 42 after infection (Figures 2A and 2B). Moreover, at day 21, when the effects of infection on splenic architecture are most pronounced, the absolute area of individual WPs was significantly smaller than in control mice and contained poorly defined T and B cell areas, with a relative paucity of T and B cells within their respective compartments (Figures 2A–2E). Features that characterized the WP after infection included the altered distribution of dendritic cells (DCs; DEC205+ CD11c+ cells). These cells are mostly restricted to the TCZ in non-infected mice, but were found throughout the WP area after infection, including in and around B cell follicles (Figures 2F and S1A).

**Figure 3:**
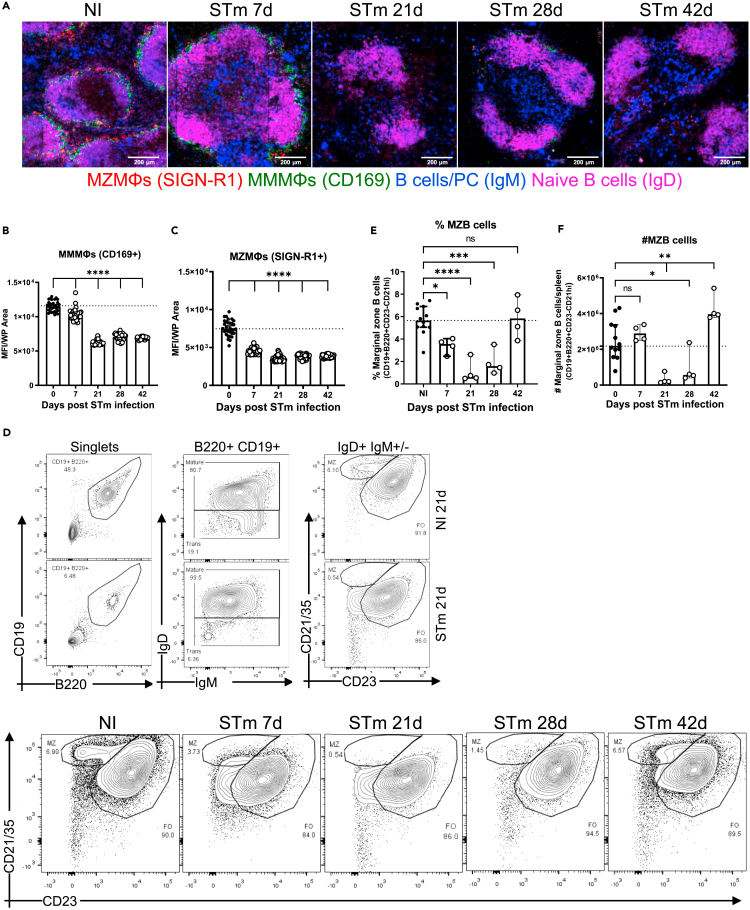
STm infection induces changes in macrophages and B cells in the MZ Mice were infected as per Figure 1. (A) Cryosections from spleens were stained to detect MMMΦs (CD169+; green), MZMΦs (SIGN-R1+; red), naive B cells (IgD+; magenta) and MZ B cells (IgM+ cells in the MZ; blue). Scale bar 200 μm. (B and C) *In situ* quantification of the signal for CD169 and SIGN-R1, respectively, in individual WP was measured and expressed as MFI. Each symbol represents the MFI per WP. (D) MZ B cells were gated on B220^+^CD19^+^IgM^+^IgD^+/−^CD21^hi^CD23^int^ expression as shown in contour plots of NI mice, and mice infected with STm for 21 days (upper panel). Representative flow cytometry contour plots for the identification of MZ B cells at different time points after infection (bottom panel). (E and F) Graphs displaying the percentage and absolute number, respectively, of MZ B cells at the indicated times. Data is representative of 2 experiments with at least 4 mice each, the bar height represents the median, and the error bars display the 95% CI. One-way ANOVA and Dunnett’s multiple comparison test was performed for B, C, E and F. ∗p< 0.05, ∗∗p< 0.01, ∗∗∗p< 0.001, ∗∗∗∗p< 0.0001. ns, nonsignificant.

The marginal zone (MZ) borders the WP and discrete populations of macrophages and B cells reside in this site, including CD169+ metallophilic macrophages (MMMΦs) and SIGN-R1+ MZ macrophages (MZMΦs) as well as MZ B cells. Moreover, the cell populations in the MZ can regulate GC B cell responses.^38^^,^^39^ By day 7 after infection, immunofluorescence (IF) microscopy showed a reduced detection of CD169+ MMMΦs, which was more apparent from day 21 and afterward, and a near absence of signal for SIGN-R1+ MZMΦs from day 7 (Figures 3A–3C and S1B–S1D). In addition, STm infection induced a reduction in B cells in the MZ as assessed by both imaging and flow cytometry (Figures 3A and 3D–3F). Although MZ B cells recovered by day 42, this was not the case for CD169+ MMMΦs and SIGN-R1+ MZMΦs (Figures S1C and S1D). Therefore, STm infection results in a significant remodeling of the WP and MZ splenic microarchitecture.

### STm-induced loss of organized FDC networks correlates with the lack of GCs

**Figure 4:**
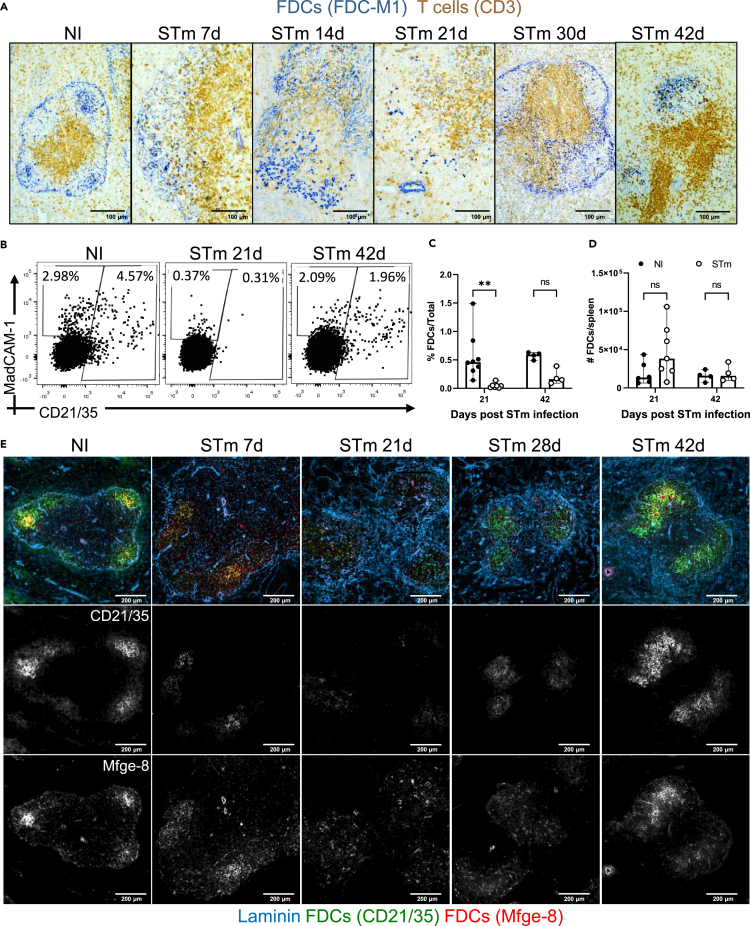
FDC network remodeling during STm infection Mice were infected as per Figure 1. (A) Representative images of spleen cryosections stained by immunohistochemistry to detect FDC (FDC-M1+; blue) and T cells (CD3^+^; brown), scale bar 100 μm. (B) Representative dot plots of FDCs and MRCs detected in spleens of NI mice and mice infected for 21 and 42 days. Cells were gated on non-haematopoietic cells (CD45), non-endothelial (CD31), non-erythroid (TER119) cells and based on the expression of podoplanin, MadCAM-1 and CD21/35 (Figure S3A). (C and D) Graphs showing the proportion of total cells and absolute number of FDCs at 21 days and 42 days after STm infection. Each point represents results from one spleen, the bar height represents the median, and the error bars display the 95% CI. two-way ANOVA and Šídák’s multiple comparison test was performed for C-D. ∗∗p< 0.01; ns, nonsignificant. (E) IF images stained to detect laminin (blue), FDCs (Mfge-8+; red), and CR1/CR2 (CD21/35+; green). Top row shows a merged image of the markers, and the middle and bottom rows show single-marker images of the same area in a grayscale. Note that B cells also express lower levels of CD21/35. Scale bar 200 μm.

**Figure 5:**
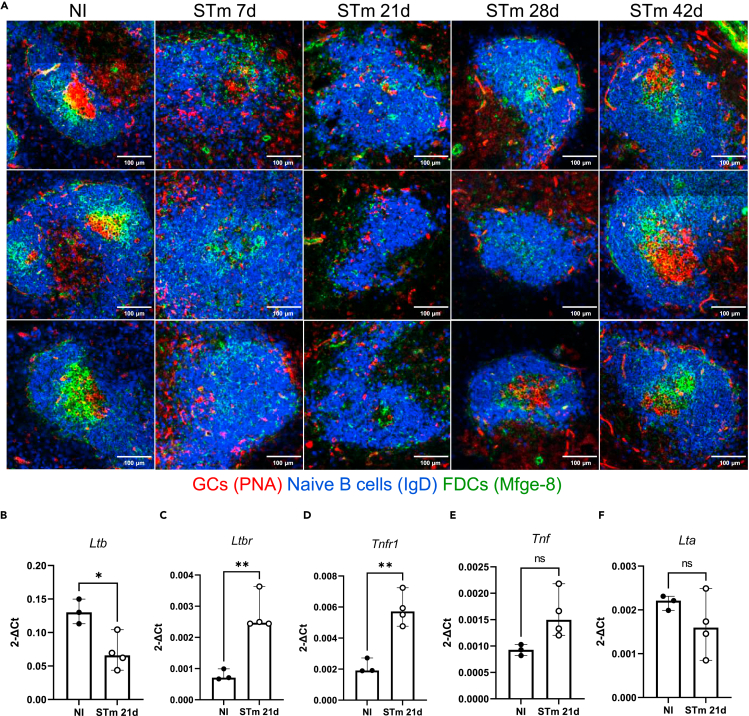
GCs are detected in parallel with the normalization of FDC networks Mice were infected as per Figure 1. (A) Representative IF images of B cell follicles (IgD+; blue), FDCs (Mfge-8+; green), and GCs (PNA+; red) detected in spleens of infected and NI mice. Three different areas of the spleen are displayed for each time point. Scale bar 100 μm. (B–F) Graphs showing the expression of individual genes in WP isolated by microdissection from NI mice or mice infected for 21 days. Each point represents the gene expression detected in WP from one individual mouse, the bar height represents the median, and the error bars display the 95% CI. Two-tailed unpaired, *t*-test was used to compare groups. ∗p< 0.05, ∗∗p< 0.01, ns, nonsignificant.

The perturbed microarchitecture observed after infection suggested that stromal cells which orchestrate the migration of cells in SLO might be affected by STm. FDCs play a critical role in the antigen-mediated selection of B cell clones in the GC,^40^ and because organized GCs are not detected in the first weeks after infection, we examined the localization of FDCs networks after infection. FDCs can be identified by the expression of milk fat globule epidermal growth factor 8 protein (Mfge-8) in conjunction with the expression of CD21/35. The classic anti-FDC antibody FDC-M1 recognizes Mfge-8.^41^ Spleens from non-infected and infected mice were stained with the FDC-M1 antibody to detect FDCs (Figure 4A). This identified a classical distribution of FDC networks in follicles and immature FDCs in the MZ in non-infected mice. Seven days after STm infection, there was a noticeable decrease in the number of compact FDC networks, instead, a filiform reticular pattern staining in the MZ predominated. By day 14 some FDC-M1+ cells were also found as individual cells distributed throughout the WP. At 21 days, few FDC-M1+ cells were detected within the follicles. Flow cytometry showed that there was a reduced density, but not absolute number of FDCs in spleens at day 21 after infection (Figures 4B–4D and S3A), reflecting the significant increase in spleen size (Figure 1B) and cellularity at this time.^42^^,^^43^ From day 28 after infection classical FDC networks started to organize again, and these appeared normal by day 42 (Figures 4A–4D and S2A). These findings were confirmed using a different IF staining panel with spleen sections co-stained for complement receptors (CR1/2; CD21/35) which are highly expressed in FDCs and with anti-Mfge-8 (18A2-G10), which target different epitopes than FDC-M1 (4C11)^41^ (Figures 4E and S2B). This confirmed the reorganization of the FDC networks and the *in situ* quantification showed a reduced MFI of FDC markers in WP (Figures S3B and S3C). This was not related to direct infection of FDC by STm as only approximately 1% of bacteria was associated with FDC at day 7 after infection and the vast majority of FDC were uninfected (Figure S3D). Next, the lack of organized FDC networks and the development of GCs were evaluated in parallel. Spleen sections from non-infected mice and STm-infected mice stained with peanut agglutinin (PNA), anti-IgD, and anti-Mfge-8. GCs (PNA+ IgD-) were only detected when classical FDC networks were recovered; for instance, at 28 days few GCs were detected only in follicles displaying a more compact FDC network and by 42 days, nearly all follicles contained a GC and an organized FDC network (Figure 5A). The maintenance of the FDC network in the adult spleen is dependent on lymphotoxin (LT)/LTβ receptor (LTβR) and tumor necrosis factor (TNF)/TNF receptor (TNFR) signaling.^44^^,^^45^^,^^46^ Gene expression of *Ltb*, *Lta*, *Ltbr*, *Tnf*, and *Tnfr1* was investigated by RT-PCR in microdissected WP isolated from the spleens of non-infected mice and mice infected for 21 days. *Ltb* expression was significantly downregulated (Figure 5B), whereas *Ltbr and Tnfr* gene expression was significantly upregulated in WP of infected mice compared to non-infected mice (Figures 5C and 5D). *Tnf* and *Lta* expression was not different between the groups (Figures 5E and 5F). Overall, the transient absence of GC is associated with the lack of FDC networks and perturbations in gene expression in the LT and TNF pathways.

### Perivascular and marginal sinus FDC precursor-like expand during infection

**Figure 6:**
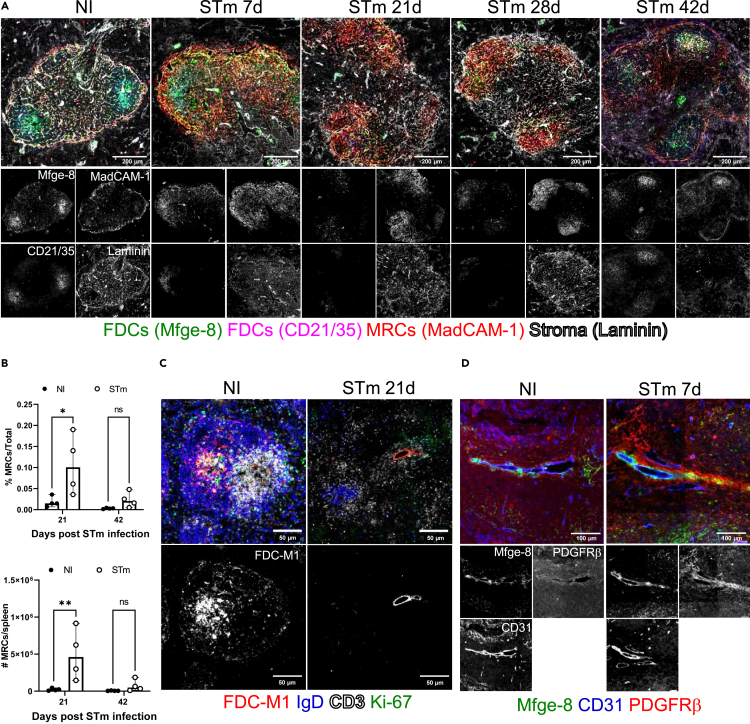
STm infection induces the expansion of MRCs Mice were infected as per Figure 1. (A) Representative IF images show MRCs (MadCAM-1+; red), FDCs (Mfge-8+; green), CR1/2 (CD21/35; magenta), and laminin (white). Top row images show all markers combined at different time points after infection. The bottom two rows of grayscale images show individual channels for each marker. Scale bar 200 μm. (B) Flow cytometry quantification of the proportion and absolute cell number of MRCs in NI mice and STm-infected mice after 21 days and 42 days. Cells were gated on CD45^−^CD31^−^TER119^−^podoplanin^+^MadCAM-1^+^CD21/35^-^ (Figure S3A). Each point represents one spleen, the bar height represents the median, and the error bars display the 95% CI. two-way ANOVA and Šídák’s multiple comparison test between the NI and STm-infected group was performed. ∗p< 0.05, ∗∗p< 0.01; ns, nonsignificant. (C) IF images on the top row show the merged staining for FDC-M1 (red), IgD (blue), CD3 (white) and Ki-67 (green). The bottom row displays single-grayscale images for FDC-M1. Scale bar 50 μm. (D) The top panels show IF images for the combined staining of Mfge-8 (green), CD31 (blue) and PDGFRβ (red). The bottom IF images show individual markers in grayscale. Scale bar 100 μm.

We assessed the effects of infection on other stromal cells that may contribute to follicle organization. One such cell-type are MRCs, which express the adhesion molecule MadCAM-1, secrete CXCL13 and have been described as marginal sinus pre-FDCs as they also share the expression of Mfge-8 in the MZ in steady-state.^14^^,^^47^ Spleen sections from non-infected mice or mice infected with STm were stained for MadCAM-1, Mfge-8, CD21/35 and laminin and assessed by IF (Figure 6A; presented as a merge of all staining on the top row or individual markers in grayscale on the bottom rows). STm infection induced a noticeable expansion of MadCAM-1+ cells from 7 days up to 28 days after the infection, and these cells were detected not only in the MZ but also in the follicles, and some were positive for Mfge-8 (Figures 6A and S4A and S4B). Analysis by flow cytometry confirmed that there was an expansion in the frequency and number of MRCs at day 21 post-infection, which was not observed at day 42 (Figures 6B and S3A). In addition, FDC-M1+/Mfge-8+ cells were observed around CD31^+^ blood vessels (Figure 6C). These cells also expressed platelet-derived growth factor receptor beta (PDGFRβ; Figure 6D) which has been described as a marker of perivascular pre-FDCs in the spleen but not in the lymph node.^47^^,^^48^^,^^49^ Therefore, MadCAM-1+ cells and perivascular PDGFRβ+ cells expand during STm infection.

### MadCAM-1-expressing cells become major producers of CXCL13 during infection

**Figure 7:**
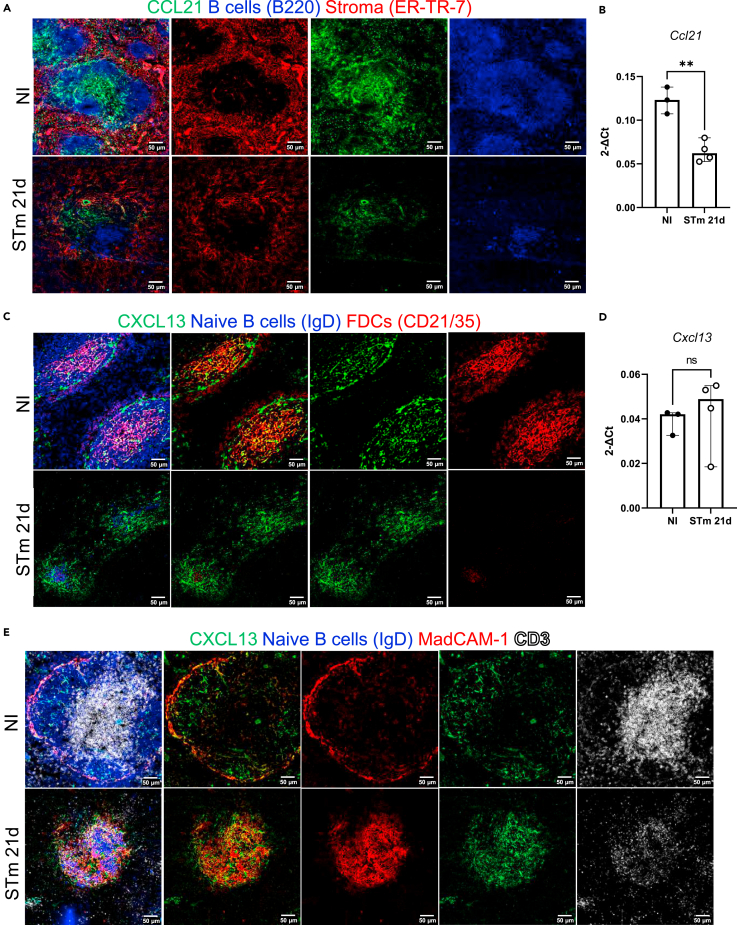
CXCL13 expression in the follicles is maintained after infection with STm Mice were infected as per Figure 1. (A) Representative IF images show CCL21 (green) expression along with B220 (blue) and ER-TR-7 (red) staining in NI mice (top row) and mice infected for 21 days (bottom row). Left-hand panels show merged images, and single-colour panels are displayed in the right-hand panels. (B) Gene expression of *Ccl21* in WP of NI mice and mice infected with STm for 21 days. (C) Representative IF images of spleen sections stained for CXCL13 (green), IgD (blue), and CD21/35 (red) in control mice (top row) and STm-infected mice for 21 days (bottom row). Merged three-color images (left), CXCL13 and CD21/35 merged images (second column), and single-color channels (two columns to the right) are displayed. Scale bar 50 μm. (D) Graph represents gene expression of *Cxcl13* in WP from NI mice and mice infected with STm for 21 days. Each point in the graphs represents the gene expression detected in WP from one individual mouse, the bar height represents the median, and the error bars display the 95% CI. Two-tailed unpaired, *t*-test was used to compare groups. ∗∗p< 0.01, ns, nonsignificant. (E) Spleen sections were stained for CXCL13 (green), IgD (blue), MadCAM-1 (red) and CD3 (white). Representative IF images on top row show NI mice and images from STm-infected mice are shown in the bottom row. Images on the right displayed four-channel merged images, MadCAM-1 and CXCL13 merged images are presented in the second column and double-positive cells are yellow. Single-color images are shown to the left. Scale bar 50 μm.

Given the reorganization of the WP and the lack of FDC networks induced after STm infection, we hypothesized that STm infection perturbs the expression of chemokine profiles within the WP. Key amongst these chemokines are CCL21 and CXCL13, which are required for normal T zone and follicle segregation and 24 h after STm infection changes in expression of these chemokines have been reported.^50^ The distribution of these chemokines was examined by IF microscopy and gene expression from microdissected WP in spleens from non-infected mice and after 21 days of STm-infection. CCL21 gene expression was reduced in the WP and this was also reflected at the protein level in T zones of infected mice compared to naive controls (Figures 7A and 7B and S5A). In contrast to early time points,^50^ and despite perturbed FDC networks being detected, the expression of CXCL13 was comparable when gene expression was measured in WP and protein expression in follicular areas (Figures 7C and 7D and S5B). In non-infected mice, most CXCL13 staining is associated with FDCs in follicles (Figures 7C and S5B). In contrast, at day 21 after infection, most CXCL13 is associated with MadCAM-1+ cells, which are distributed throughout the WP and are not restricted to the marginal sinus (Figures 7E and S5C). Thus, during STm infection MadCAM-1+ cells provide an alternative source of CXCL13 to compensate for the reduced expression of this chemokine by FDCs during this time.

## Discussion

Here, we show how systemic *Salmonella* infection induces the disorganization of the splenic WP and loss of GC formation, and the later detection of GCs correlates with the induction and resolution of these changes. The capacity of STm infection to impact GC induction and maintenance is not restricted to STm-specific responses but also impacts GC induced to a diverse spectrum of other pathogens and antigens, including model antigens, bacterial flagellin, the microbiota, influenza virus and the helminth *Nippostrongylus brasiliensis*.^32^^,^^33^^,^^34^^,^^37^ The diversity of these antigens and the observation that STm infection impacts pre-existing and ongoing GC responses indicates the antigen independence of these effects. Because B cells from STm-infected mice maintain the capacity to develop into GC B cells,^37^ it indicates that a significant contributory reason for the absence of GC in the first weeks of infection is that STm disrupts the niches in which GCs develop.

Previous studies have shown how non-B cell-intrinsic mechanisms including the recruitment of discrete Sca-1+ monocyte populations, TLR4 expression and IL-12-mediated suppression of Tfh cells can impair GC formation after STm infection.^34^^,^^37^ The impact of IL-12 induced to *Salmonella* is likely to act early, possibly in the first 24 h after infection because this cytokine is produced by conventional and monocyte-derived dendritic cells from 2 h post-infection to promote Th1 differentiation.^33^ In contrast to these early events, the identification of perturbed FDC and MZ organization occurs later and indicates that the structures needed to support GC development and function are not maintained during the peak of the infection. Moreover, MZ B cells, MMMΦs and MZMΦs, which support GC responses,^38^^,^^39^ were detected less readily after day 7 after infection when GCs were absent. Unexpectedly, only MZ B cells were found at levels comparable to non-infected mice when GCs developed. The two macrophage populations had not returned to normal by then, therefore, more work is needed to understand the potential role of MZ populations in GC responses after STm infection. Despite the multiple and quite marked effects of *Salmonella* on the host adaptive immune response, it is important to contextualize the lack of GCs as being a selective effect and not representative of a general impairment in the capacity of the host to induce antibody responses to the pathogen. Many B cell responses remain active in the host during the period when GC are not detectable. For instance, extensive EF B cell responses are detectable from the first days after infection.^24^ IgM responses are induced in a T-independent manner whereas B cell switching to IgG dependent upon BCL6^+^PD1^lo^CXCR5^lo^ T cells and CD40L.^24^^,^^51^^,^^52^ Moreover, antibodies derived from the EF response can moderate bacteremia and subsequent re-infection.^24^ Therefore, the tissue reorganization observed in primary infection is not a barrier to productive EF responses, and so the impairment of the GC response to *Salmonella* is unlikely to have evolved simply as a strategy to impair antibody responses *per se*. Therefore, whether there is a selective advantage to the host or to the pathogen remains unclear. In contrast to the lack of GCs observed after live infection, GC responses are detected within the first four days after immunization with outer membrane vesicles (OMVs) from STm.^53^ These OMVs contain many of the immunodominant cell surface antigens from STm, including LPS and porins.^54^ This rapid induction of Tfh cells and GCs^53^ suggests that exposure of the host to multiple STm antigens in their natural conformation within the bacterial membrane, concomitant with significant TLR4 ligating activity, is not sufficient to explain why GCs are not induced to this pathogen.

A hallmark of the GC is the organization of multiple cell types within follicles. This includes FDCs which are essential not only for providing chemokines to recruit B cells but also for antigen-driven selection.^13^^,^^19^^,^^55^ The STm-induced changes in FDCs and the downregulation of key phenotypic markers, alongside the timing of these changes, is fully consistent with changes in FDCs being a key reason why GC do not form or are not maintained. Nevertheless, it is unlikely that direct infection of FDCs drives these effects as bacteria are rarely detected in FDC and only infrequently in B cell follicles. Bacteria mainly localize within iNOS+ inflammatory foci in the red pulp at the times examined here.^56^^,^^57^ This contrasts with what is observed after other infections where impaired GC responses associated with disruption of FDCs have been observed. For instance, FDCs are directly infected and subsequently lost in draining LNs after the Bluetongue virus infection of sheep.^58^ GC development requires the complement component C3^59^ and this factor has also been shown to be important for the localization of pneumococcal bacteria to FDCs.^60^ The role of complement in the context of GCs and *Salmonella* infections is not well explored, and in the presence of antibodies (which are induced rapidly in this infection^24^), C3 from mice is not deposited as efficiently as human C3 on the surface of *Salmonella in vitro*.^61^ This may also partially explain why STm were only rarely associated with FDCs, or there may be other reasons such as the rate of bacteria capture by phagocytic cells. Therefore, how complement may contribute, or otherwise, to GC formation during STm infection requires further investigation.

FDCs are plastic stromal cells that originate from perivascular cells and require B cell-derived TNFR and LTβR signaling for their maintenance.^45^^,^^46^^,^^47^^,^^62^^,^^63^^,^^64^^,^^65^ The de-clustering of FDC induced by STm infection is unlikely to reflect the death of FDCs as although the density of FDCs in the spleen was significantly reduced after infection, the total numbers were not and these cells are known to be long-lived and resistant to stresses such as radiation exposure.^13^^,^^16^^,^^40^ Another potential outcome is that FDCs lose maturity and partially de-differentiate as FDC-like cells were readily detectable in the follicles and expansion of MadCAM-1+ MRCs in the MZ and throughout the follicle was observed, possibly because of exposure to TLR4 ligands.^66^ Likewise, the capacity to form normal FDC networks could also occur because of the reduced density of B cells in the follicles or/and an intrinsic decrease of LTαβ expression, which may limit LTβR signaling. The other LTβR ligand, LIGHT (*TNFSF14*) may contribute to this process.^67^ Here, we have shown that the expression of genes within the TNFR and LTβR signaling pathways in the WP were modulated by STm infection. TNF is produced by multiple innate immune cell types and T cells after infection and plays pleiotropic roles in controlling infection in tissues such as the spleen and liver.^68^^,^^69^^,^^70^ Less is known about the contribution of LT and TNF to the organization of WP after STm infection. We did attempt to modulate FDCs by targeting the LTβR and modulation of TNF using agonistic and neutralizing antibodies, respectively. Targeting LTβR did not provide consistent results, with some mice showing accelerated induction of FDCs and GCs, but not all. The reasons for this are unclear but we speculate that they may be consequences of trying to maintain agonistic LTβR signaling for a sufficiently long time to have lasting effects. In contrast, neutralizing TNF during *E*. *muris* infection enhances GC responses^25^ but we observed an enhanced loss of FDCs and no GC response during STm-infection. The loss of FDCs, GCs and impaired humoral immunity after interference with the TNF signaling using either TNF-neutralizing antibodies or gene-targeted mice has been described extensively in steady-state conditions^46^^,^^64^^,^^65^^,^^71^ and under inflammatory environments such as sepsis.^72^ These results suggest that during active infection, the targeting of specific cell types may be more difficult than targeting soluble factors such as TNF and highlights the potential difficulty in using single agent interventions to target different bacterial infections as each infection may have a distinct immune signature.

### Limitations of the study

A limitation of this study is the lack of formal demonstration that changes in FDC organization are responsible for the lack of early GC induction and other mechanistic insights. Probably, there are multiple active pathways that contribute to the suppression of GCs during Salmonella infection, and simply moderating inflammation may not be sufficient to account for these effects. Furthermore, we have not demonstrated the function of individual stromal cellular subsets that we have described. For some cell types, such as FDC precursors and MRCs, the relationship between surface marker expression, anatomical location and function is an area of active investigation. It is possible that activities associated with these cell types may change as more studies are published. Moreover, we have not examined the cellular makeup for MZ macrophages, at times significantly beyond day 42 when GC responses become established.

## STAR★Methods

### Key resources table

**Table:** 

REAGENT or RESOURCE	SOURCE	IDENTIFIER
**Antibodies**		

Unconjugated Rat monoclonal anti-CD16/32 (93)	Thermo Fisher Scientific	Cat# 14-0161-82; RRID:AB_467133
Alexa Fluor® 488 Rat anti-B220 (RA3-6B2)	BD Bioscience	Cat# 557669; RRID:AB_396781
BD Horizon™ BV786 Rat monoclonal anti-CD19 (1D3)	BD Biosciences	Cat# 563333; RRID:AB_2738141
Brilliant Violet 421™ Rat monoclonal anti-IgD (11–26c.2a)	BioLegend	Cat# 405725; RRID:AB_2562743
APC-eFluor™ 780 Rat monoclonal anti-IgM (II/41)	Thermo Fisher Scientific	Cat# 47-5790-82; RRID:AB_2573984
Alexa Fluor® 647 Rat monoclonal anti-CD21/35 (7E9)	BioLegend	Cat# 123424; RRID:AB_2629578
PE Rat monoclonal anti-CD23 (B3B4)	BD Biosciences	Cat# 553139; RRID:AB_394654
Alexa Fluor™ 488 Rat monoclonal anti-CD45 (30-F11)	Thermo Fisher Scientific	Cat# 53-0451-82; RRID:AB_2848416
Alexa Fluor® 700 Rat monoclonal anti-erythroid cells (TER-119)	BD Biosciences	Cat# 560508; RRID:AB_1645211
PE Syrian Hamster anti-Podoplanin (8.1.1)	Thermo Fisher Scientific	Cat# 12-5381-82; RRID:AB_1907439
BV605 Rat monoclonal anti-CD31 (390)	BD Biosciences	Cat# 740356; RRID:AB_2740088
BD OptiBuild™ BV786 Rat monoclonal anti-MadCAM-1 (MECA-367)	BD Biosciences	Cat# 742817; RRID:AB_2741069
Purified Hamster monoclonal anti-CD3e (145-2C11)	BD Biosciences	Cat# 553058; RRID:AB_394591
Purified Rat monoclonal anti-IgD (11–26c.2a)	BD Biosciences	Cat# 553438; RRID:AB_394858
Purified Rat monoclonal anti-follicular dendritic cell (FDC-M1)	BD Biosciences	Cat# 551320; RRID:AB_394151
Purified Rat monoclonal anti-MadCAM-1 (MECA-367)	BioLegend	Cat# 120702; RRID:AB_493393
Purified Rat monoclonal anti-B220 (RA3-6B2)	Thermo Fisher Scientific	Cat# 14-0452-86; RRID:AB_467256
Purified Rabbit polyclonal anti-Laminin	Thermo Fisher Scientific	Cat# PA1-16730; RRID:AB_2133633
Purified Armenian hamster monoclonal anti-CD209b (SIGN-R1; 22D1)	Thermo Fisher Scientific	Cat# 14-2093-82; RRID:AB_795885
Purified Rat monoclonal anti-CD169 (Siglec-1; 3D6.112)	BioLegend	Cat# 142402; RRID:AB_10916523
APC Rat monoclonal anti-CD205 (205yekta)	Thermo Fisher Scientific	Cat# 17-2051-82; RRID:AB_1548730
Purified Armenian Hamster anti-CD11c (HL3)	BD Biosciences	Cat# 553799; RRID:AB_395058
Alexa Fluor 555 Goat anti-IgM (Polyclonal)	SouthernBiotech	Cat# 1020–32; RRID:AB_2794223
Purified Hamster anti-milk fat globule epidermal growth factor 8 protein (Mfge-8; 18A2-G10)	MBL International	Cat# D199-3; RRID:AB_590479
Purified Rat anti-fibroblasts monoclonal antibody (ER-TR-7)	Thermo Fisher Scientific	Cat# MA1-40076; RRID:AB_1074409
Biotin Recombinant monoclonal anti-CD21/35 (REA800)	Miltenyi Biotec	Cat# 130-111-650; RRID:AB_2656312
Purified Goat anti-CD31/PECAM-1 (Polyclonal)	R&D System	Cat# AF3628; RRID:AB_2161028
PE-eFluor™ 610 Rat monoclonal anti-PDGFRβ (APB5)	Thermo Fisher Scientific	Cat# 61-1402-82, RRID:AB_2815310
Purified Goat anti-CCL21 (Polyclonal)	R&D System	Cat# AF457; RRID:AB_2072083
Purified Goat anti-CXCL13 (Polyclonal)	R&D System	Cat# AF470; RRID:AB_355378
Purified Rat monoclonal anti-MadCAM-1 (MECA-367)	BioLegend	Cat# 120702; RRID:AB_493393
Purified Rabbit anti-Ki-67 (Polyclonal)	Abcam	Cat# ab15580; RRID:AB_443209
Purified Rabbit anti-*Salmonella* (Polyclonal)	Abcam	Cat# ab35156; RRID:AB_777811
Peroxidase Goat anti-Armenian Hamster IgG (H + L)	Fitzgerald Industries International	Cat# 43R-IG098HRP; RRID:AB_1286595
Biotin-SP-AffiniPure Donkey Anti-Rat IgG (H + L)	Jackson ImmunoResearch Labs	Cat# 712-065-153; RRID:AB_2315779
Peroxidase-AffiniPure Donkey Anti-Rat IgG (H + L)	Jackson ImmunoResearch Labs	Cat# 712-035-153; RRID:AB_2340639
Alexa Fluor 488-AffiniPure Donkey Anti-Rat IgG (H + L)	Jackson ImmunoResearch Labs	Cat# 712-545-153; RRID:AB_2340684
Cy3-AffiniPure Donkey Anti-Rat IgG (H + L)	Jackson ImmunoResearch Labs	Cat# 712-165-153; RRID:AB_2340667
Alexa Fluor 647-AffiniPure Goat Anti-Armenian Hamster IgG (H + L)	Jackson ImmunoResearch Labs	Cat# 127-605-160; RRID:AB_2339001
Cy3-AffiniPure Donkey Anti-Rabbit IgG (H + L)	Jackson ImmunoResearch Labs	Cat# 711-165-152; RRID:AB_2307443
DyLight 405-AffiniPure Donkey Anti-Rabbit IgG (H + L)	Jackson ImmunoResearch Labs	Cat# 711-475-152; RRID:AB_2340616
Alexa Fluor 488-AffiniPure Donkey Anti-Sheep IgG (H + L)	Jackson ImmunoResearch Labs	Cat# 713-545-147; RRID:AB_2340745
Alexa Fluor 488 Polyclonal Antibody	Thermo Fisher Scientific	Cat# A-11094; RRID:AB_221544
Alexa Fluor 488-AffiniPure Donkey Anti-Rabbit IgG (H + L)	Jackson ImmunoResearch Labs	Cat# 711-545-152; RRID:AB_2313584

**Bacterial and virus strains**		

*aroA*-deficient *S*. Typhimurium SL3261 attenuated strain	Cunningham et al.^24^	

**Chemicals, peptides, and recombinant proteins**		

Dulbecco’s Phosphate-Buffered Saline (DPBS)	Thermo Fisher Scientific	Cat# 14190094
Luria-Bertani (LB) Broth with agar (Lennox), Powder microbial growth medium	Sigma-Aldrich	Cat# L2897-1 KG
LB Broth (Lennox)	Sigma-Aldrich	Cat# L3022-1 KG
RPMI-1640 Medium	Thermo Fisher Scientific	Cat# 21875034
Fetal Bovine Serum (FBS)	Thermo Fisher Scientific	Cat# 10500064
Ethylenediaminetetraacetic acid (EDTA) solution pH 8.0 (0.5 M)	AppliChem	Cat# A4892,0100
Ammonium-chloride-potassium (ACK) lysing buffer	Thermo Fisher Scientific	Cat# A1049201
Collagenase P	Roche	Cat# 11213857001
DNAase I	Roche	Cat# 10104159001
3,3′-Diaminobenzidine (DAB) tablets 10 mg	Sigma-Aldrich	Cat# D5905
Hydrogen peroxide solution	Sigma-Aldrich	Cat# H-1009-500ML
Naphthol AS-MX Phosphate-free acid	Sigma-Aldrich	Cat# N4875
Fast Blue BB Salt hemi(zinc chloride) salt	Sigma-Aldrich	Cat# F3378
Levamisole [(−)-tetramisole hydrochloride	Sigma-Aldrich	Cat# L9756
VectaMount Permanent Mounting Medium	Vector Laboratories	Cat# H-5000-60
Pro-Long Glass Antifade Mountant	Thermo Fisher Scientific	Cat# P36984
Peanut Agglutinin (PNA), Biotinylated	Vector Laboratories	Cat# B-1075-5
AlexaFluor 555-conjugated Streptavidin	Thermo Fisher Scientific	Cat# S32355
AlexaFluor 488-conjugated Streptavidin	Thermo Fisher Scientific	Cat# S32354
Brilliant Violet 421-conjugated Streptavidin	BioLegend	Cat# 405225
Fixable viability dye eFluor 450	Thermo Fisher Scientific	Cat# 65-0863-14
UltraComp eBeads™ Plus Compensation Beads	Thermo Fisher Scientific	Cat# 01-3333-42
Multispot Microscopy Slides	Hendley-Essex	Cat# A051121 PH-314

**Critical commercial assays**		

CD45 MACS microbeads, mouse	Miltenyi Biotec	Cat# 130-052-301; RRID:AB_2877061
TER119 MACS microbeads, mouse	Miltenyi Biotec	Cat# 130-049-901
LS Columns	Miltenyi Biotec	Cat# 130-042-401
VECTASTAIN[R] ABC-AP Kit	Vector Laboratories	Cat# AK-5000; RRID:AB_2336792
RNeasy Micro kit	Qiagen	Cat# 74004
High-capacity cDNA Reverse Transcription Synthesis Kit	Thermo Fisher Scientific	Cat# 4368813

**Experimental models: Organisms/strains**		

Mouse: C57BL/6 mice	Charles River Laboratory	Strain Code: 027

**Software and algorithms**		

FlowJo Software v10.8.1 for Windows	BD Bioscience	RRID:SCR_008520; https://www.flowjo.com
GraphPad Prism 9	Version 9.4.1 (681)	https://www.graphpad.com
ZEISS Axio Scan.Z1 Slide Scanner	Zeiss	RRID:SCR_020927
ImageJ	NIH	RRID:SCR_003070; https://imagej.nih.gov/ij/
Black Zen software	Zeiss	RRID:SCR_018163; https://www.zeiss.com/microscopy/en/products/software/zeiss-zen.html
PALM Robo Software V.4.6	Zeiss	RRID:SCR_014435
SDS software (SDS 2.3)	Applied Biosystems	RRID:SCR_014596
Biorender	Biorender	RRID:SCR_018361; https://biorender.com

### Resource availability

#### Lead contact

Further information and requests for resources and reagents should be directed to and will be fulfilled by the lead contact, Adam F. Cunningham (a.f.cunningham@bham.ac.uk).

#### Materials availability

This study did not generate new unique reagents.

### Experimental model and subject details

#### Systemic infection in mice

All the experiments were performed following the animal research regulations established by the Animals (Scientific Procedures) Act 1986 (ASPA) and under the licenses, P06779746 and I01581970 issued by the Home Office. Wild-type adult C57BL/6 mice (6–8 weeks, specific-pathogen-free males) were purchased from Charles River Laboratory and housed in the Biomedical Services Unit at the University of Birmingham during the infection period. Mice were systemically infected (intraperitoneally) with 5 × 10^5^ colony-forming units (CFU) of *aroA*-deficient *S*. Typhimurium SL3261 attenuated strain as previously described.^24^^,^^57^ Control mice were injected with sterile-filtered Dulbecco’s-phosphate-buffered saline (DPBS). Spleens were collected at the indicated time points after the infection. Bacteria load per organ was quantified by plating 10-fold diluted tissue homogenates on Luria-Bertani agar plates.

### Method details

#### Flow cytometry

Single-cell suspensions were obtained by mechanical disaggregation of the spleen across 50 μm-cell strainers and washed with cold RPMI-1640 medium containing 5% fetal bovine serum (FBS) and 5 mM ethylenediaminetetraacetic acid (EDTA). Red blood cells were lysed using ammonium-chloride-potassium (ACK) lysing buffer (Life Technologies). Cells suspensions were incubated with a CD16/32 antibody (eBioscience) in FACS buffer (2% FBS, 5 mM EDTA and 0.01% of sodium azide in PBS) to reduce FcγRIII/FcγRII-mediated non-specific binding. Fixable viability dye eFluor 450 (eBioscience) was used to discriminate between live and dead cells. Cell suspensions were incubated with a mix of the following antibodies in FACS buffer: anti-B220 (RA3-6B2; BD Bioscience), anti-CD19 (1D3; BD Bioscience), anti-IgD (11–26c.2a; BioLegend), anti-IgM (II/41; eBioscience), anti-CD21/35 (7E9; BioLegend) and anti-CD23 (B3B4; BD Bioscience). To quantify stromal populations, a portion of spleens was digested on a solution containing 0.2 mg mL^−1^ collagenase P (Roche), 0.1 mg mL^−1^ DNAase I in RPMI-1640 medium and enriched by depleting CD45^+^ cell and TER119+ cells using MACS microbeads (Miltenyi Biotec). Cells suspensions were incubated with anti-CD45 (30-F11; eBioscience), anti-erythroid cells (TER-119; BD Bioscience), anti-Podoplanin (8.1.1; eBioscience), anti-CD31 (390; BD Bioscience), anti-MadCAM-1 (MECA-367; BD Bioscience) and anti-CD21/35 (7E9; BioLegend). Samples were acquired using a BD LSR II Fortessa flow cytometer and data was analyzed using Flowjo Software v10.8.1 for Windows (Ashland, OR, USA).

#### Immunohistochemistry

Spleens were obtained at different time points after infection as indicated in each figure and frozen in liquid nitrogen. Five-μm slices were obtained using a Bright cryostat and fixed with acetone at 4°C for 20 min. Slides were rehydrated in Tris Buffer (TBS; pH 7.6) and labeled with the following antibodies for immunohistochemistry analysis: anti-CD3 (145-2C11; BD Bioscience), anti-IgD (11–26c.2a; BD Bioscience), anti-follicular dendritic cell (FDC-M1; BD Bioscience), anti-CD209b (SIGN-R1; 22D1; eBioscience), anti-CD169 (Siglec-1; 3D6.112; BioLegend), and anti-B220 (RA3-6B2). The following secondary antibodies were used and incubated at room temperature for an hour: Peroxidase-conjugated goat anti-Armenian Hamster IgG (H + L) from Fitzgerald, Biotin-SP-AffiniPure Donkey Anti-Rat IgG (H + L) and Peroxidase-AffiniPure Donkey Anti-Rat IgG (H + L) from Jackson ImmunoResearch (Cambridge, UK). Peroxidase activity was detected using SIGMAFAST3,3′-Diaminobenzidine (DAB) tablets and hydrogen peroxide dissolved in TBS buffer pH 7.6. Alkaline phosphatase enzyme activity was detected using VECTASTAIN[R] ABC-AP Kit (Vector Laboratory) and developed with a mix of Naphthol AS-MX Phosphate-free acid, fast blue B salt and Levamisole [(−)-tetramisole hydrochloride] in TBS buffer pH 9.2. All chemical reagents for immunohistochemistry were purchased from Sigma-Aldrich. Slides were mounted in VectaMount Permanent Mounting Medium (Vector Laboratories), and the total spleen area was imaged using a microscope slide scanner (Zeiss Axio Scan.Z1).

#### Immunofluorescence microscopy

Cryosections were rehydrated in PBS-0.01% Tween 20 and incubated at room temperature with the primary antibodies in a PBS solution containing 1% of BSA, 1% of normal human serum and 0.01% of sodium azide. The following antibodies were used: anti-IgD (11–26c.2a; BD Bioscience), anti-B220 (RA3-6B2), anti-CD3 (145-2C11; BD Bioscience), anti-laminin (Polyclonal; Invitrogen), anti-CD209b (SIGN-R1; 22D1; eBioscience), anti-CD169 (Siglec-1; 3D6.112; BioLegend), anti-CD205 (205yekta; eBioscience), anti-CD11c (HL3; BD Biosciences), anti-IgM (Polyclonal; Southern Biotech), anti-milk fat globule epidermal growth factor 8 protein (Mfge-8; 18A2-G10; MBL International), anti-fibroblasts monoclonal antibody (ER-TR-7; Thermo Fisher Scientific), anti-follicular dendritic cell (FDC-M1; BD Bioscience), anti-CD21/35 (REA800; Miltenyi Biotec), anti-CD31 (Polyclonal; R&D System), anti-PDGFRβ (APB5; Thermo Fisher Scientific), anti-CCL21 (Polyclonal; R&D System) and anti-CXCL13 (Polyclonal; R&D System), anti-MadCAM-1 (MECA-367; eBioscience), anti-Ki-67 (Polyclonal; Abcam), peanut agglutinin (PNA; Vector Laboratories), and anti-*Salmonella* (Polyclonal; Abcam). The following fluorescent-labelled streptavidin and secondary antibodies were used to detect unconjugated antibodies and incubated for 45 min in the dark: AlexaFluor 488-conjugated anti-rat IgG, Cy3-conjugated anti-rat IgG, AlexaFluor 647-conjugated anti-hamster IgG, Cy3-conjugated anti-rabbit IgG, DyLight 405-conjugated anti-rabbit IgG, AlexaFluor 555-conjugated streptavidin, AlexaFluor 488-conjugated streptavidin, Brilliant Violet 421-conjugated streptavidin. Four-step indirect IF staining was performed for the detection of chemokines using AlexaFluor 488-conjugated anti-sheep IgG, rabbit anti-AlexaFluor 488, and AlexaFluor 488-conjugated anti-rabbit IgG. Slides were mounted with Pro-Long Glass Antifade Mountant (Thermo Fisher Scientific), and images were acquired with Zeiss Axio Scan.Z1 or Zeiss LSM780 Confocal Microscope. Pixel intensity quantification was performed with Fiji (ImageJ) software (National Institutes of Health) and ZEN 3.0 Software. The absolute area of GCs was quantified by manually selecting PNA-positive clusters in IgD-negative areas inside follicles.

#### Laser capture microdissection, RNA isolation and quantitative PCR

Eight-micron sections for microdissection were cut onto membrane slides (Carl Zeiss 1.0 PEN NF 41590-9081-000), stained with 1% (v/v) cresyl violet acetate and stored at −80°C. Slides were bought to room temperature and microdissection was performed using the PALM Robo Software V.4.6 using the brightfield “AxioCam CC1” setting on a Zeiss PALM Micro-Beam Laser Capture Microdissection microscope. Microdissected tissue was collected into tubes containing 20 μL of RLT buffer and β-Mercaptoethanol. Afterward, collection tubes were stored at −80°Cprior to RNA extraction. RNA extraction from the microdissected samples was completed as per the protocols provided using the RNeasy micro kit (Qiagen). The RNA was then reverse transcribed using the high-capacity reverse transcription cDNA synthesis kit (Applied Biosystems) according to the manufacturer’s specifications. Quantitative RT-PCR (Applied Biosystems) was performed on cDNA samples for *Ccl19*, *Cxcl13*, *Lta*, *Ltb*, *Ltbr*, *Tnfr1* and *Tnfa* mRNA expression. β-actin was used as an endogenous control. The primers and probes used were from Applied Biosystems, and samples were run in duplicates on a 384-well PCR plate (Applied Biosystems) and detected using an ABI PRISM 7900HT instrument. Results were analyzed with the Applied Biosystems SDS software (SDS 2.3). The mean of two technical replicates (C_t_ values) was used to calculate the ΔC_t_ value for which the C_t_ of the β-actin was subtracted from the C_t_ of the target gene C_t_ value, and the relative amount was calculated as 2^−Δ^^C^_t_. C_t_ values above 34 were not accepted, and neither were technical replicates with more than two-cycle differences between them.

#### Quantification and statistical analysis

Statistical analysis was performed using GraphPad Prism 9 for Windows version 9.4.1 (681). The bar height in all graphs represents the median, and the error bars display the 95% confidence interval (CI). Two-tailed, unpaired, *t*-test was applied when comparing two groups, and one-way ANOVA and Tukey’s or Dunnett’s multiple comparison test was performed when comparing between groups or against the non-infected (NI) control, respectively. A significant difference between groups was considered when the pvalue was <0.05.

## Data Availability

Data reported in this paper will be shared by the lead contact upon request. This paper does not report original code. Any additional information required to reanalyse the data reported in this paper is available from the lead contact upon request.
