# Supplementary material for: Salmonella infection induces the reorganization of follicular dendritic cell networks concomitant with the failure to generate germinal centers
Source: iScience. 2023 Mar 2;26(4):106310. doi: 10.1016/j.isci.2023.106310 (PMC10025972; doi:10.1016/j.isci.2023.106310)
Supplement: Document S1. Figures S1–S5 [file mmc1.pdf]

iScience, Volume *26*

Supplemental information

Salmonella infection induces the reorganization of follicular dendritic cell networks concomitant with the failure to generate germinal centers

Edith Marcial-Juárez, Marisol Pérez-Toledo, Saba Nayar, Elena Pipi, Areej Alshayea, Ruby Persaud, Sian E. Jossi, Rachel Lamerton, Francesca Barone, Ian R. Henderson, and Adam F. Cunningham

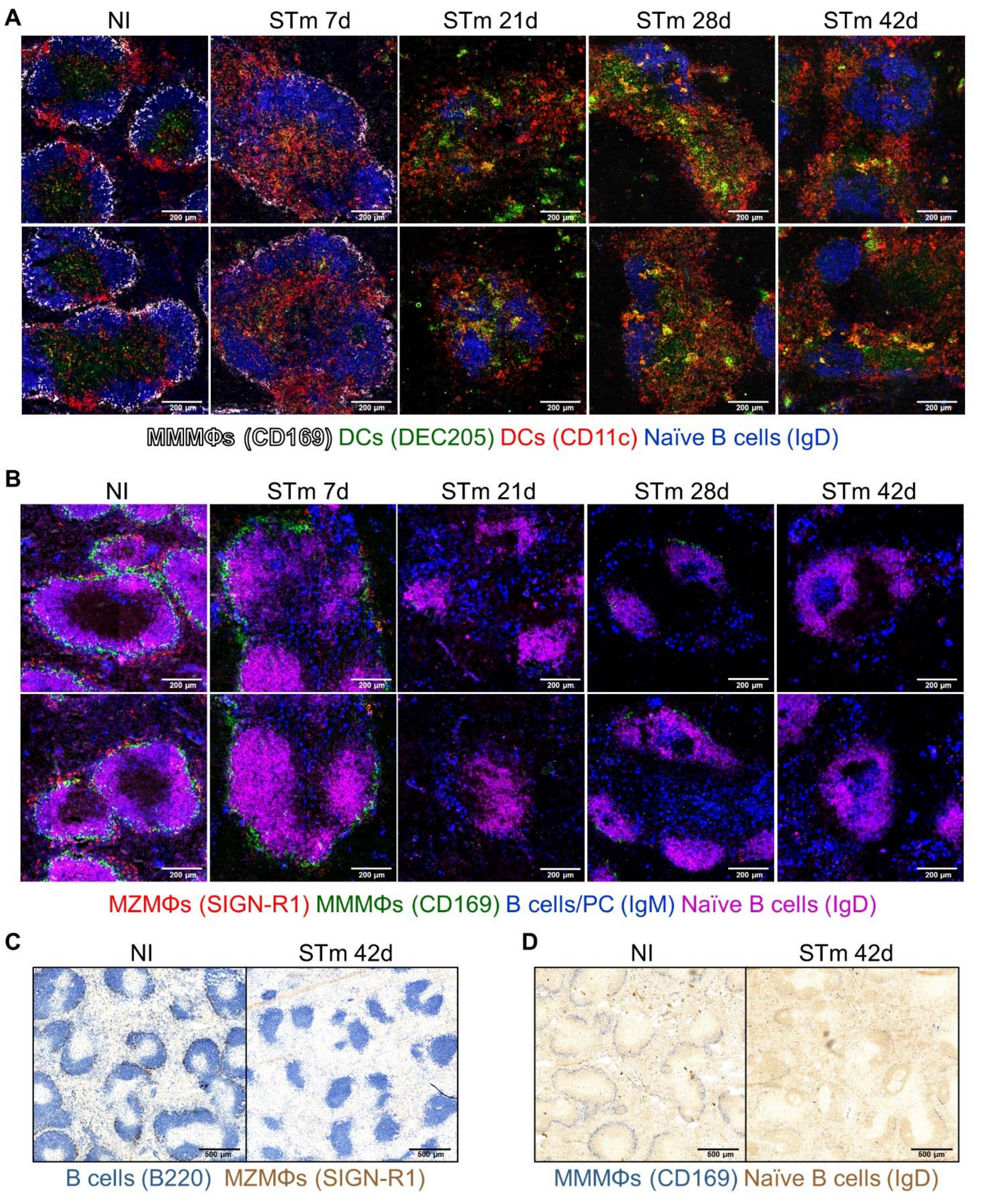

Figure S1. DCs, MZMΦs, MMMΦs and MZ B cells in the spleen after STm infection, related to Figures 2 and 3. Mice were infected as per Figure 1. **(A)** Cryosections from spleens were stained to detect MMMΦs (CD169+; white), DCs (DEC205+; green), CD11c+ (red), and naïve B cells (IgD+; blue). Scale bar 200 µm. **(B)** Cryosections from spleens were stained to detect MZMΦs (SIGN-R1+; red), MMMΦs (CD169+; green), MZ B cells (IgM+ cells in the MZ; blue) and B cells (IgD+; magenta). Scale bar 200 µm. **(C)** Representative low-magnification images of spleen cryosections stained by immunohistochemistry to detect B cells (B220+; blue) and MZMΦs (SIGN-R1+; brown) in non-infected (NI) mice and STm-infected mice for 42 days. Scale bar 500 µm. **(D)** Representative low-magnification images of spleen cryosections stained by immunohistochemistry to detect MMMΦs (CD169+; blue) and naïve B cells (IgD+; brown) in NI mice and STm-infected mice for 42 days. Scale bar 500 µm.

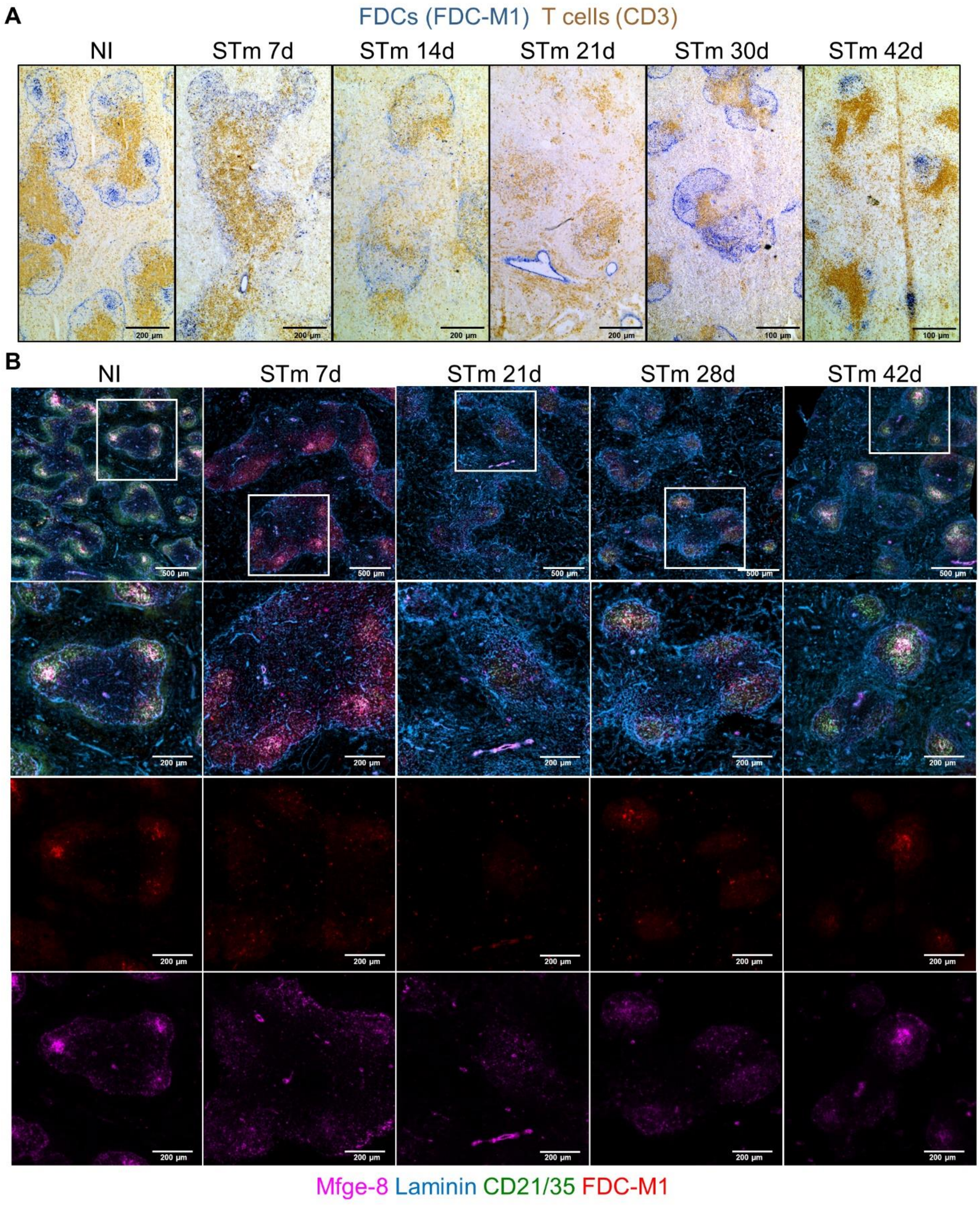

Figure S2. FDC networks analysis in the spleen during STm infection, related to Figure 4. Mice were infected as per Figure 1. **(A)** Representative images of spleen cryosections stained by immunohistochemistry to detect FDC (FDC-M1+; blue) and T cells (CD3+; brown). Scale bar 200 µm. **(B)** IF images stained to detect laminin (blue), FDCs (Mfge-8+; magenta and FDC-M1; red), and CR1/CR2 (CD21/35+; green). Top row shows low magnification and merged images of all markers, and the second row represents higher magnifications of the selected areas. The bottom two rows show single-colour images. Scale bar 200 µm.

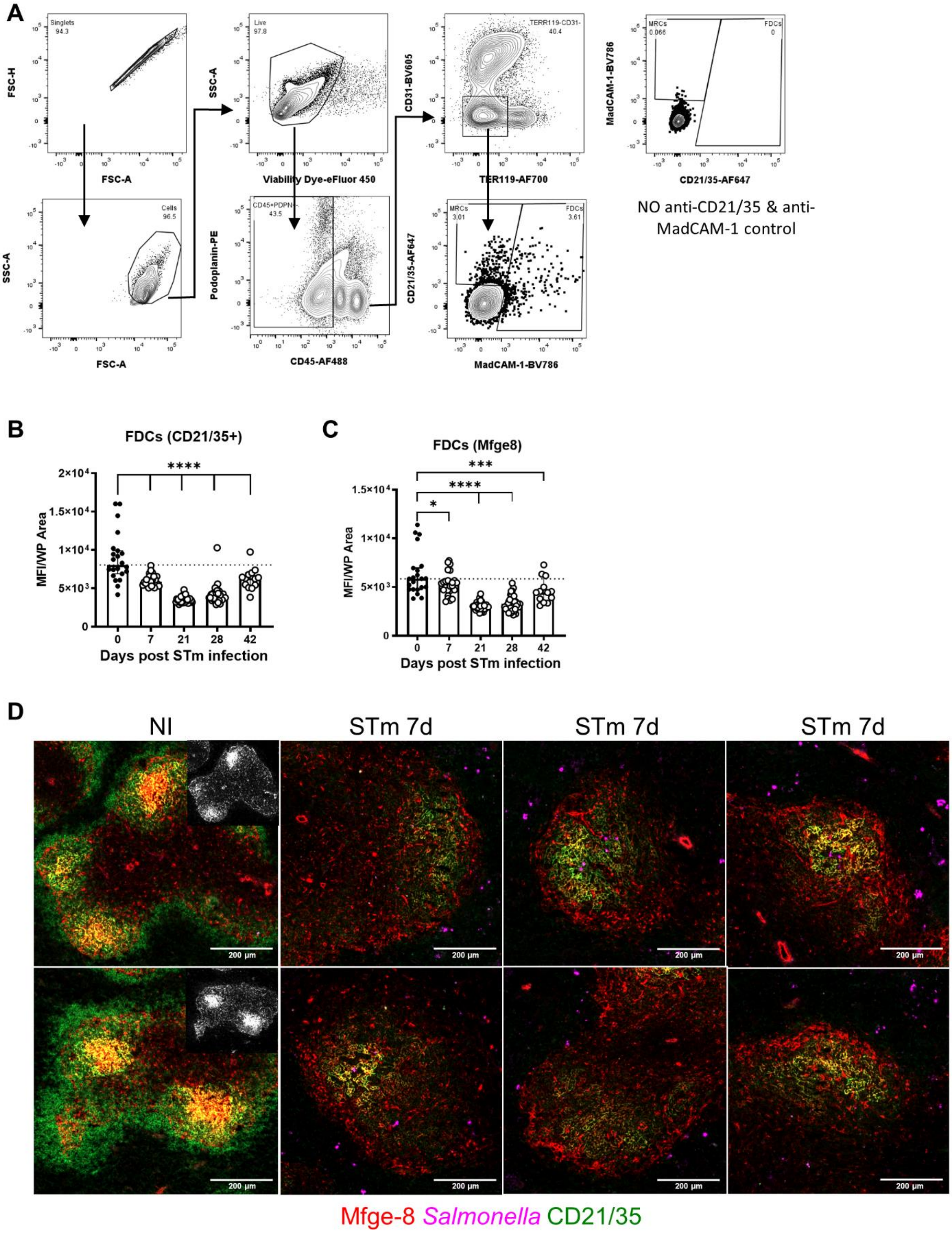

Figure S3. Gating strategy for the analysis of stromal cells in the spleen and distribution of STm *in situ*, related to Figures 4 and 6. Mice were infected as per Figure 1. **(A)** Cell suspensions were obtained by enzymatic digestion and depletion of CD45+ cells, and TER119+ cells was performed using MACS microbeads. Cell suspensions were stained with anti-CD45, anti-CD31, anti-TER119, anti-podoplanin, anti-MadCAM-1, anti-CD21/35, and the viability dye-eFluor 450. Representative contour plots are shown to display the gating strategy for the analysis of FDCs and MRCs. The plot to the right represents a control sample containing all the antibodies except anti-CD21/35 and anti-MadCAM-1. **(B, C)** Graphs display the *in situ* quantification of the MFI for the detection of CD21/35 and Mfge-8 at different time points after STm infection in individual WP. Each symbol represents the MFI per WP, the bar height represents the median, and the error bars display the 95% CI. One-way ANOVA and Dunnett's multiple comparison test was performed for **B** and **C**. *p < 0.05, ***p < 0.001, ****p < 0.0001. **(D)** Cryosections were stained to detect FDCs (Mfge-8+; red), CR1/2 (CD21/35+; green)

and STm (magenta) in NI mice (two images to the left) and mice infected for 7 days (six images to the right). The yellow signal represents Mfge-8 and CD21/35 double positive cells. Grayscale images in NI control display single staining of Mfge-8. Scale bar 200 µm.

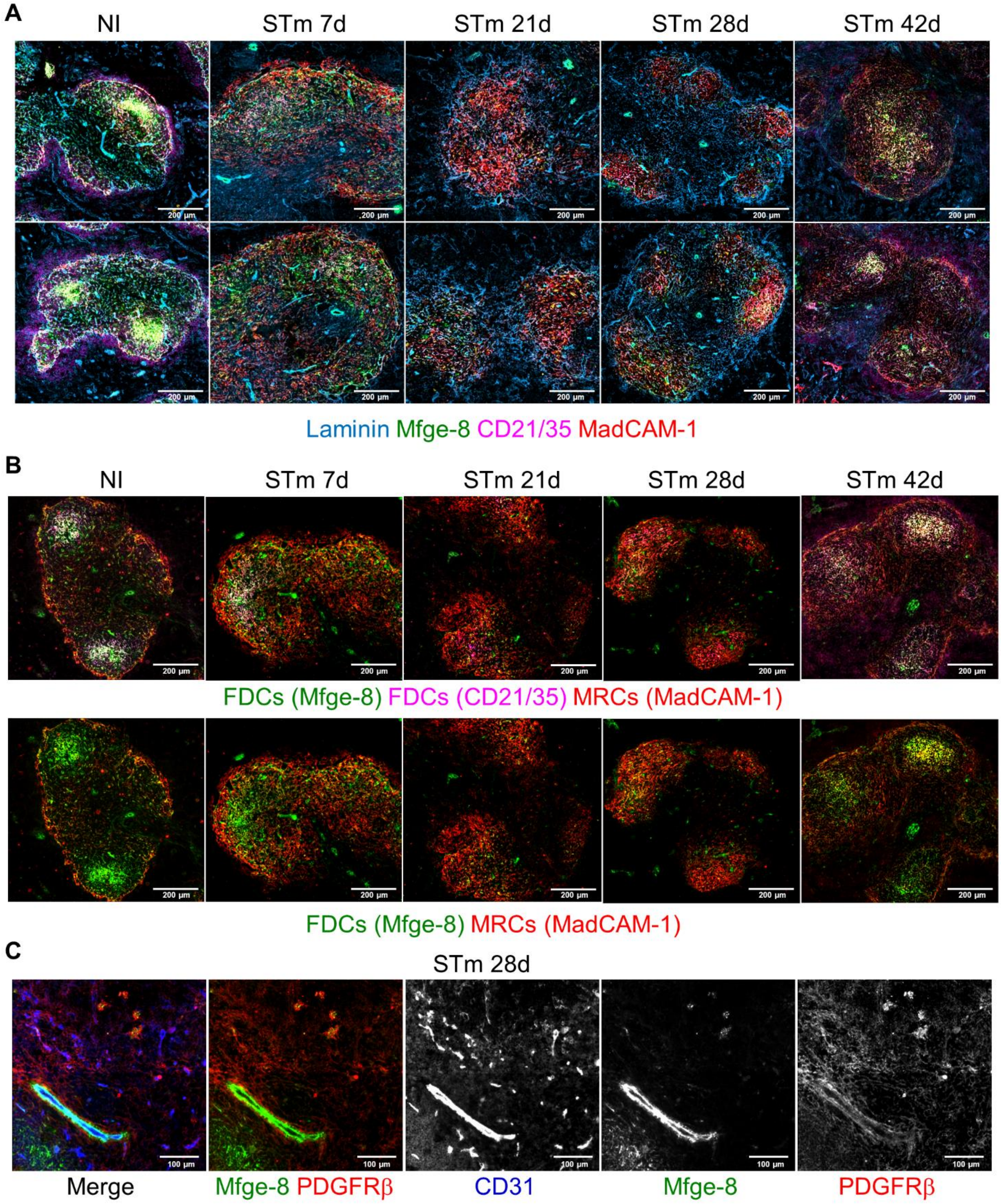

Figure S4. FDCs and MRCs in the WP of the spleen after STm infection, related to Figure 6. Mice were infected as per Figure 1. **(A)** Representative IF images (two different areas of the spleen) show MRCs (MadCAM-1+; red), FDCs (Mfge-8+; green), CR1/2 (CD21/35; magenta), and laminin (blue). Scale bar 200 µm. **(B)** Representative IF images show MRCs (MadCAM-1+; red), FDCs (Mfge-8+; green) and CR1/2 (CD21/35; magenta). Top row images show the three markers combined at different time points after infection; triple positive cells are white. The bottom row shows the merge of MadCAM-1 and Mfge-8 signal; double positive cells are yellow. Scale bar 200 µm. **(C)** IF images show triple staining of Mfge-8 (green), CD31 (blue) and

PDGFRβ (red), double staining of Mfge-8 (green) and PDGFRβ (red) and individual markers (grayscale) in the spleen of STm infected mice after 28 days. Scale bar 100 µm.

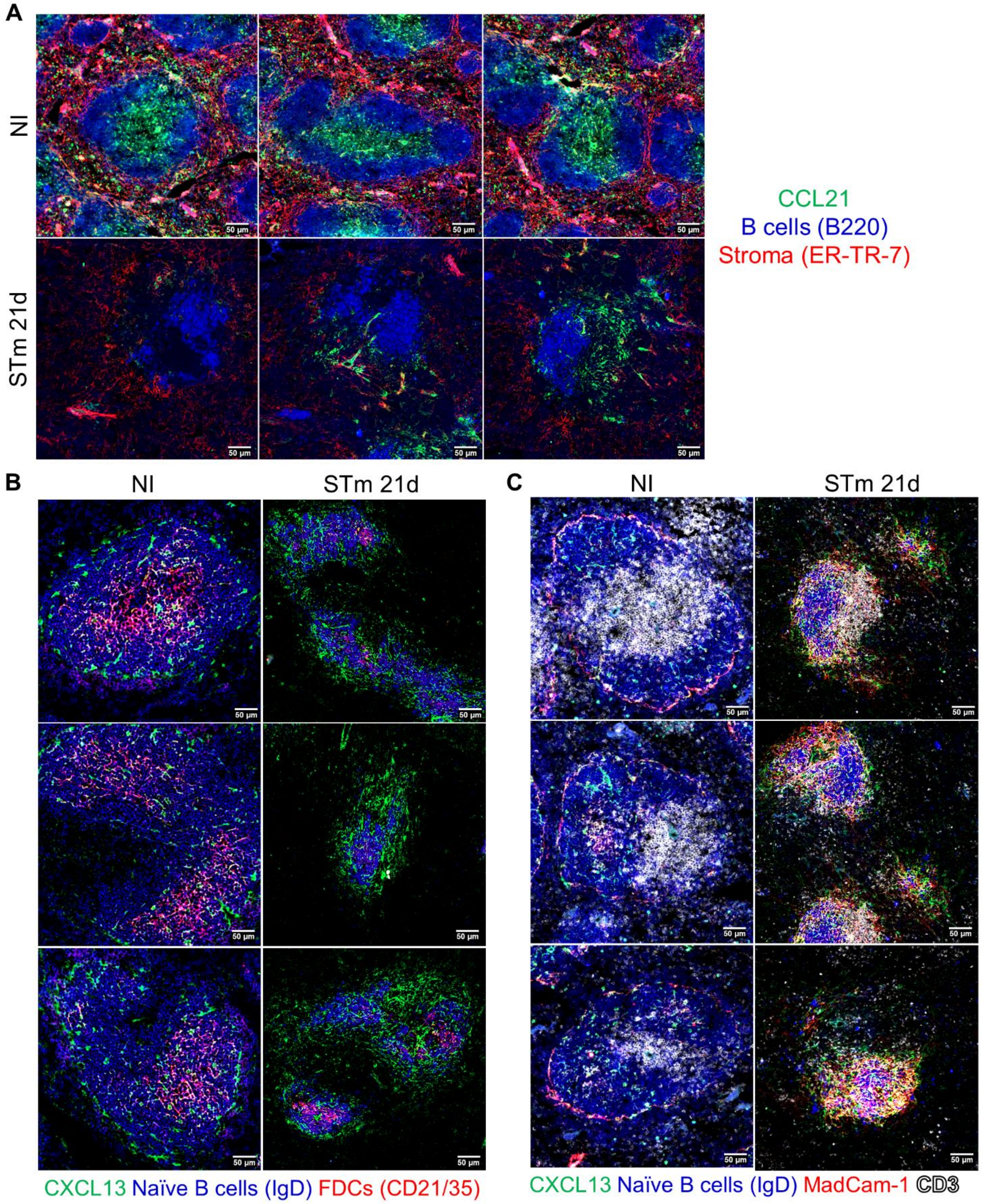

Figure S5. Chemokine expression in the splenic WP after STm infection, related to Figure 7. Mice were infected as per Figure 1. **(A)** Representative IF images show CCL21 (green) expression along with B220 (blue) and ER-TR-7 (red) staining in NI mice (top row) and mice infected for 21 days (bottom row). Scale bar 50 µm. **(B)** Representative IF images of spleen sections stained for CXCL13 (green), IgD (blue), and CD21/35 (red) in control mice (left) and mice infected with STm for 21 days (right). Scale bar 50 µm. **(C)** Spleen sections from NI mice (left) and STm-infected mice (right) were stained for CXCL13 (green), IgD (blue), MadCAM-1 (red) and CD3 (white). Scale bar 50 µm.
